# Targeting protein kinase CK2 suppresses bladder cancer cell survival via the glucose metabolic pathway

**DOI:** 10.18632/oncotarget.13571

**Published:** 2016-11-24

**Authors:** Xiaolei Zhang, Xiao Yang, Chengdi Yang, Peng Li, Wenbo Yuan, Xiaheng Deng, Yidong Cheng, Pengchao Li, Haiwei Yang, Jun Tao, Qiang Lu

**Affiliations:** ^1^ Department of Urology, The First Affiliated Hospital of Nanjing Medical University, Nanjing 210029, China

**Keywords:** bladder cancer, CK2α, glycolysis, oncogene, prognosis

## Abstract

Casein kinase 2 (CK2) is a constitutively active serine/threonine kinase that promotes cell proliferation and resists apoptosis. Elevated CK2 expression has been demonstrated in several solid tumors. The expression of CK2α in bladder cancer was elevated in tumor tissues compared with that in adjacent normal tissues. Amplified expression of CK2α was highly correlated with histological grade in bladder cancer(P = 0.024). Knockdown of CK2α in bladder cancer cell lines resulted in a reduction in tumor aerobic glycolysis, accompanied with lower phosphorylated AKT. Moreover, low CK2α levels suppressed cell growth, and similar results could be reproduced after treatment with CX-4945 with a dose-dependent response. CX-4945 inhibited migration and induced apoptosis. Furthermore, knockdown of CK2α decreased the tumorigenicity of bladder cancer cells in vivo. This study is the first to report that CK2 increases glucose metabolism in human bladder cancer. Blocking CK2 function may provide novel diagnostic and potential therapeutic.

## INTRODUCTION

Bladder cancer was the sixth most commonly diagnosed cancer in males in 2012 worldwide and the most common malignancy of the urogenital tract. In 2012, 429,800 new cases of bladder cancer were reported worldwide, meanwhile 165,100 patients died from the disease [1].

Protein kinase casein kinase 2 (CK2) is a conserved, ubiquitously expressed protein serine/threonine kinase that was first discovered by Burnett and Kennedy in 1954 [2]. CK2 exists primarily as a holoenzyme consisting of two catalytic subunits, CK2α and CK2α’, and two β regulatory subunits. Functionally, CK2 is involved in the modulation of major cellular processes and pathways [3, 4]. Subsequent studies have shown that CK2 was a potent regulator in the processes of cell proliferation, differentiation, and apoptosis, which was involved in numerous signaling pathways including PI3K/AKT, NF-κB, Wnt, Notch1 and Hedgehog/Gli1 [5–9]. Although its pro-survival function is necessary for the whole organism, it is ‘more necessary’ for cancer cells than normal. Elevated level of CK2α has been observed in numerous cancers, including breast [10], prostate [11], lung [12, 13], head and neck [14], colorectal [15], gastric [16], and kidney [17]. These evidences validated that CK2 could be a potential cancer therapeutic target. CX-4945 was the first effective and selective CK2 inhibitor used in human clinical trials. CX-4945 inhibits the activity of CK2α and CK2α’, resulted to suppression of activation of pro-survival signaling pathways and promotion of apoptosis in human glioblastoma, breast, prostate, and lung cancers [13, 18–20].

Normal cells metabolize glucose to produce ATP by oxidative phosphorylation and a large amount of pyruvate which is oxidized via the tricarboxylic acid cycle. The rate of production is coupled with oxygen respiration and proton transport [21]. By contrast, tumor cells consume glucose to produce lactate and support mitochondrial oxidative phosphorylation even in oxygen-rich conditions. This phenomenon is referred to as “aerobic glycolysis” or the “Warburg effect” which was first observed in the 1930s by Otto Warburg [22]. Increased glycolysis is widely considered as a crucial hallmark of cancer, which has been consistently observed in many cancer cell lines [23]. Recent studies indicated that cellular metabolism was directly or indirectly linked to activated oncogenes and inactivated cancer suppressors [23, 24]. The expression level of glycolysis-related genes, such as glucose transporter (GLUT)1, lactic dehydrogenase (LDH)A, LDHB, hexokinase (HK)1, HK2, and pyruvate kinase type M (PKM), are elevated in certain cancer tissues [25], which have been observed in our previous work. Thus, enhanced dependency of cancer cells on glycolysis probably provides a biochemical basis to kill malignant cells preferentially than normal cells by inhibiting glycolysis.

Above all, we reported the function of CK2α in bladder cancer and further investigated the possible relationship between CK2α and glucolysis. We also tested the potential anticancer effect of inhibition of CK2 in vitro and vivo, suggesting its possible application in the treatment of bladder tumors.

## RESULTS

### Overexpression of CK2α in human bladder cancer

**Figure 1:**
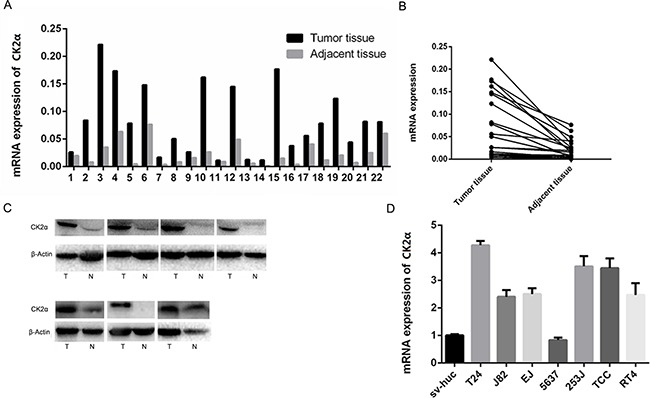
Expression level of CK2α mRNA and protein in human primary bladder cancer cell lines and surgical specimens as evaluated **A, B**. RT-qPCR showed that relative *CK2α* mRNA expression in 22 BCa and adjacent tissues (*P* < 0.05). **C**. CK2α protein in seven BCa and adjacent tissues (N, non-tumor, T, tumor). **D**. CK2α mRNA levels was up-regulated in seven bladder cancer cell lines compared with those in the normal urinary epithelial cell line sv-Huc.

Primary paired BCa tissue samples and BCa cell lines were used to examine CK2α expression. The gene expression of CK2α was analyzed by qRT-PCR in 22 pairs of BCa tissues and their adjacent tissues. Compared with adjacent bladder tissues, the mRNA level of CK2α was significantly overexpressed in the bladder tumor tissues (P < 0.01; Figure 1A and 1B). To investigate whether differences in expression of mRNA level are reflected at the protein level, Western blot analysis was conducted. Consistent with the PCR results, CK2α protein expression was significantly higher in the BCa tissues compared with that in the adjacent tissues (Figure 1C). qRT-PCR analysis demonstrated that mRNA level of CK2α in six bladder cancer cell lines (T24, J82, EJ, 253J, TCC, and RT4) was increased (particularly in T24) compared with that in the normal urinary epithelial cell line sv-Huc (Figure 1D). Thus, CK2α expression was positively associated with bladder cancer progression, suggesting that it played an oncogenic role in bladder cancer.

### IHC analysis of CK2α expression in BCa clinical samples

**Figure 2:**
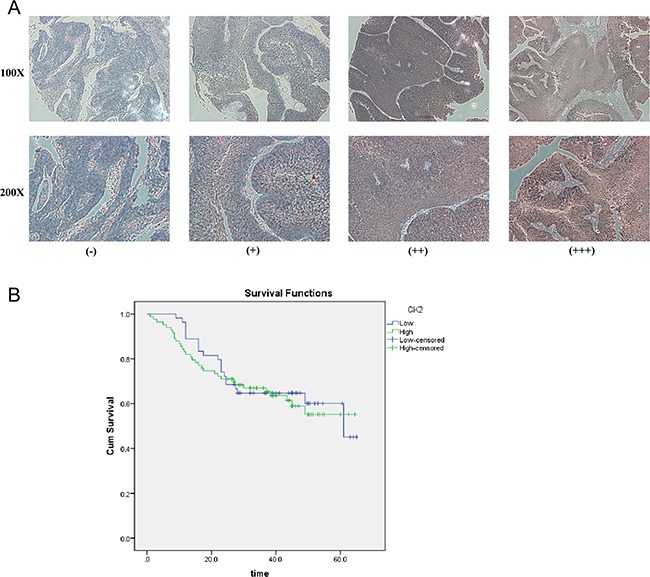
IHC analyses of CK2α protein expression in primary BCa surgical specimens and Kaplan–Meier survival analyses of the primary BCa patients (n = 137) **A**. CK2α staining in BCa, scored as CK2α (-) (+) (++) (+++). All images are shown at × 100 magnification and × 200 magnification. **B**. BCa patients were divided into low-CK2α expression (*n* = 54, CK2α- or CK2α+) and high-CK2α expression (*n* = 83, CK2α++ or CK2α+++) groups. Survival analyses did not reveal a significant association between high CK2α expression and poor prognosis (*P* = 0.694, log-rank test).

To further explore the role and prognostic value of CK2α in human bladder cancer, TMAs with 160 cases of bladder tumor samples were used to examine CK2α expression via IHC. In the CK2α-positive specimens, high CK2α expression (++ or +++) was found in 101 (63.1%) specimens, and low CK2α expression (− or +) was detected in 59 (36.9%) specimens (Table 1). Correlations between the clinicopathological parameters of BCa and expression of CK2α are summarized in Table 1. Chi-square analyses revealed that CK2α expression was positively correlated with histological grade (P = 0.024) but not with tumor size and tumor stage (TNM). Kaplan–Meier analyses did not reveal a significant association between high CK2α expression and poor prognosis(P = 0.694; Figure 2B).

**Table 1:** Correlation between CK2α expression and clinicopathological variables of 160 patients with bladder cancer

Clinicopathologic variable	CK2α expression			
	N	Low	High	P
All cases	160	59	101	
Age(years)				0.373
<65	74	30	44	
≥65	86	29	57	
Gender				0.970
Male	125	46	79	
Female	35	13	22	
TNM stage				0.308
Stage I	92	37	55	
Stage II and III	68	22	46	
Histological grade				
grade I and II	79	36	43	0.024*
grade III	81	23	58	
Tumor size(cm)				0.611
<3	110	42	68	
≥3	50	17	33	

* *P* < 0.05

### CK2α promotes bladder cancer cell proliferation

**Figure 3:**
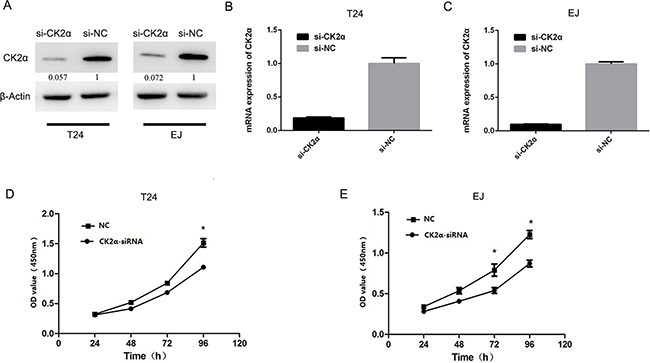
CK2α was essential for bladder cancer cell proliferation **A, B, C**. Knockdown efficiency of selected CK2α-targeting siRNAs in transfected cells was evaluated by Western blot and qRT-PCR. **D, E**. CCK8 assay showed that silencing of CK2α suppressed proliferation of T24 (D) and EJ (E) cell lines.

Cell proliferation assay was conducted to explore the role of CK2α in the growth of bladder cancer cells. T24 and EJ cells were evaluated in cell proliferation assays after they were transiently transfected with CK2α-specific siRNAs and siNC RNA (negative control, NC) for 48 h,. Cell proliferation (P < 0.05; Figures 3D and 3E) was significantly inhibited in T24 and EJ cells transiently transfected with siCK2α compared with those transfected with siNC. These results supported that CK2α promoted the growth of BCa cells.

### Inhibition of CK2α downregulates the glucose metabolic pathway

**Figure 4:**
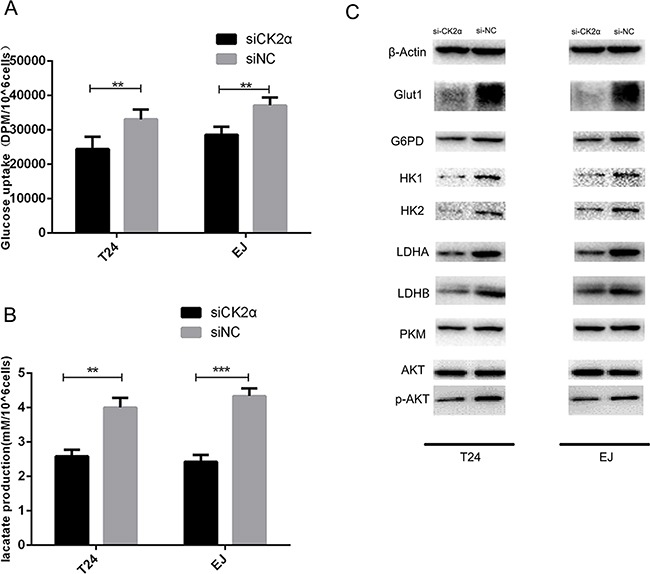
CK2α inhibition induced a metabolic shift in bladder cancer cells **A**. Glucose uptake assays. CK2α inhibition decreased glucose uptake. **B**. Lactate production assays. CK2α inhibition suppressed lactate secretion. **C**. CK2α inhibition decreased protein expression in cancer aerobic glycolysis.

**Figure 5:**
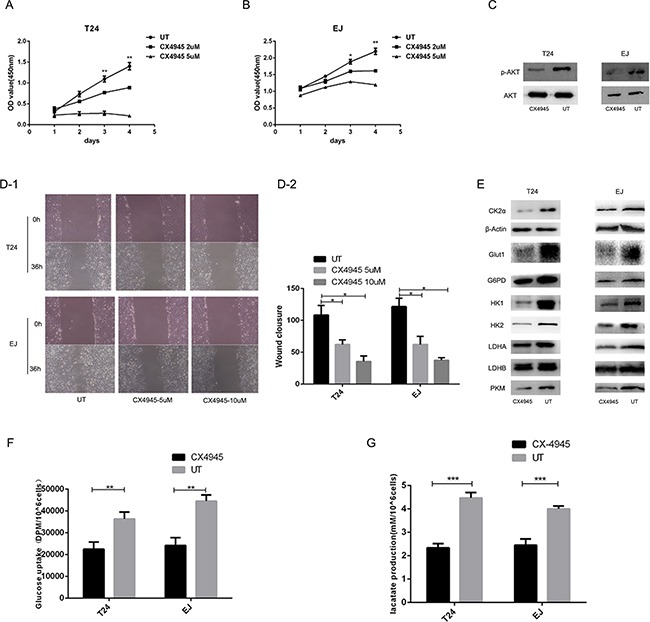
Effects of inhibition of CK2 on bladder cancer cell function **A, B**. CX-4945 suppressed proliferation of T24 (A) and EJ (B) cell. **C, E**. Western blot analysis showed that protein expression in cancer aerobic glycolysis was downregulated in T24 and EJ cells after treatment with CX4945. AKT and p-AKT(ser473) (C). GLUT1, G6PD, HK1, HK2, LDHA, LDHB and PKM (E). **D**. CX-4945 inhibited the migration ability in T24 and EJ cell lines. **F**. Glucose uptake assays. CX-4945 decreased glucose uptake. **G**. Lactate production assays. CX-4945 suppressed lactate secretion.

To investigate whether CK2 suppression influences the glucose metabolic pathway, we silenced CK2α expression. At 48 h after transfection, the efficiency of RNA interference was monitored by Western blot and qRT-PCR. The corresponding expression of CK2α in T24 and EJ cell lines was decreased (Figures 3A, 3B, and 3C). Glucose uptake and lactate secretion in bladder cancer cells decreased when CK2α was inhibited (Figures 4A and 4B). AKT was found to be less phosphorylated at Ser473 in CK2α-silenced cells than in control cells (Figure 4C). AKT is considered the Warburg kinase. PI3K/AKT/mTOR signaling links several tumor cell metabolic processes, including glycolysis. As such, we found that the protein level of glycolysis-related genes, including GLUT1, G6PD, LDHA, LDHB, HK1, HK2, and PKM, was lower in CK2α-silenced cells compared with those in control cells (Figure 4C). Furthermore, treatment with CX-4945 suppressed glucose uptake and lactate secretion (Figures 5F and 5G) in bladder cancer cells. AKT was less phosphorylated at Ser473, and the protein level of glycolysis-related genes was lower in cells after treatment with CX4945 (Figures 5C and 5E). Taken together, CK2α inhibition may suppress high glycolysis levels via suppressing AKT phosphorylation in bladder cancer cells.

### Effects of inhibition of CK2 on BCa cell function

**Figure 6:**
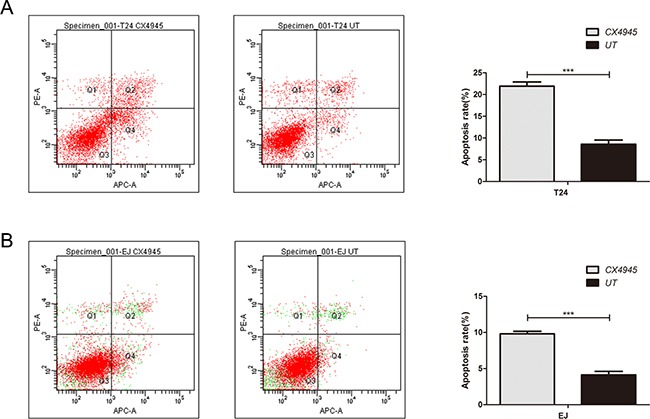
Effect of inhibition of CK2 on apoptosis in bladder cancer cells CX-4945 induced apoptosis in T24(A) and EJ(B) cell lines.

Treatment with CX-4945 suppressed bladder cancer cell growth in a dose-dependent manner (Figures 5A and 5B). We found that treatment with CX-4945 significantly inhibited the migration ability in bladder cancer cells (Figure 5D; both P < 0.05). CX-4945 induced apoptosis in both cell lines (Figure 6; both P < 0.05). Thus, CK2 inhibition abrogated many biological functions of bladder cancer that are critical to tumor growth and survival.

### Knockdown of CK2α inhibits the growth of tumor xenografts in nude mice

**Figure 7:**
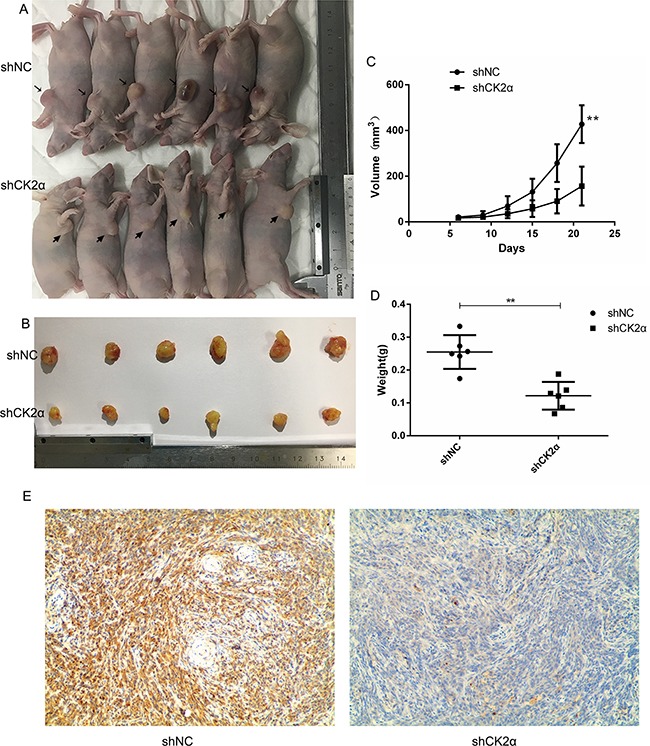
CK2α inhibition decreased tumorigenesis and reduced cancer cell growth in vivo **A, B**. CK2αknockdown EJ cells formed smaller tumour volume compared to the control cells (NC). **C**. The tumor growth curves in the two groups (*P* < 0.01). **D**. CK2αknockdown reduced tumor weight compared to the control cells (NC) (*P* < 0.01). **E**. CK2α immunohistochemistry staining of xenografted tumors retrieved from treated nude mice. Representative images are shown. (magnification, ×100)

We evaluated the effects of CK2α Knockdown on tumor growth in vivo. The transfected EJ cells were implanted into the right axilla of the nude mouse by subcutaneous injection, and the tumor volumes were measured after injection. Compared with the control cells, the mean volumes of the xenograft tumors treated with CK2α knockdown cells showed obvious growth retardation (Figure 7A, 7B, 7C, 7D). Immunohistochemical staining identified that the level of CK2α was inhibited in the CK2α knockdown tumors (Figure 7E).

## DISCUSSION

Protein kinase CK2α, one isoform of the catalytic subunit of serine/threonine protein kinase CK2, has been demonstrated to be overexpressed in various malignancies [26–28]. However, its underlying role in bladder cancer remains unclear.

In this study, we revealed that CK2α was frequently and significantly upregulated in human bladder cancer. The level of CK2α in bladder cancer cell lines except 5637 was increased compared with that in the normal urinary epithelial cell line sv-Huc. The difference results might be caused by low malignance of 5637 or heterogeneity of tumor. We expect further research on this issue. IHC analyses convincingly demonstrated that CK2α was overexpressed in primary bladder cancer. Increased expression of CK2α was shown in 63.1% of BCa samples, and was significantly correlated with tumor grade. These findings indicated that CK2α was an oncogene promoting bladder cancer progression and correlated with pathogenesis.

As a prognostic marker in various malignancies, CK2α expression is positively correlated with histological grade, distant metastasis, tumor stage and outcome [15, 27, 29]. However, we found no significant evidence to identify the relationship between high CK2α expression and tumor stage and prognosis. These differences might be attributed to tumor heterogeneity.

Numerous signaling pathways have been researched, through which CK2 regulates cancer cell survival, but the relationship between CK2 and glucose metabolism remains unknown. Our present work suggested that CK2 may be a positive regulator in cell survival via the glucose metabolic pathway in bladder cancer. Several lines of evidence support this finding. Firstly, the inhibition of CK2α resulted in the downregulation of glycolysis-related protein level, as well as decrease in glucose uptake and lactate production. Secondly, suppressed cell proliferation was observed after CK2α knockdown. Finally, similar results were reproduced after treatment with CX-4945 even with a dose-dependent response in both cell lines.

Our previous study demonstrated that the expression level of the glycolysis-related genes in bladder tumor tissues was frequently overexpressed compared with those in adjacent tissues [25]. Through the glucose metabolic pathway, tumor cells consume glucose to produce a large number of ATP, lactate, NAD+, NADPH, and H+ cofactors, which are used for the growth and proliferation of cancer cells. In our study, we knocked down CK2α level in bladder cancer cells and then observed decrease in glucose uptake and lactate production, accompanied with low level of GLUT1, HK, PKM, and LDH. These results suggested that CK2α positively regulated the activation of glycolysis. Interestingly, CK2α silencing reduced the expression level of G6PD, which was of great importance for the pentose phosphate pathway (PPP). PPP is a key source of NADPH, as a cofactor in maintaining the redox balance and for anabolic pathways, such as fatty acid biosynthesis [30]. Moreover, PPP produces ribose and is involved in the transcription of gene expression during stress conditions [31].

In human glioblastoma, CK2 suppresses apoptosis and promotes oncogenes via enhancing the AKT pathways and downstream gene expression [18]. Consistent with these observations, AKT was found to be less phosphorylated in CK2α knockdown cells than in control cells, which suggested that CK2α-regulated glycolysis was related to the AKT pathway. Several reports conveyed the ability of CK2 inhibitors to suppress the PI3K-AKT-mTOR pathway [32–34]. Dr. Ruzzenne demonstrated that CK2 phosphorylates AKT directly at Ser129, further increasing the catalytic activity of AKT [35]. Unsurprisingly, hyper activation of PI3K-AKT-mTOR signaling was shown to be a consequence of CK2 overexpression [36, 37]. Researchers have found that the PI3K-AKT-mTOR axis was an essential pathway for various cellular processes, including glycolysis [38, 39]. Moreover, AKT has been reported to function as a master regulator of energy metabolism in cancer cells by enhancing the expression of glycolytic regulators [38]. Taken together, CK2α inhibition may suppress high glycolysis level via the CK2α/AKT-mTOR cascade in bladder cancer cells.

Notably, bladder cancer cell treatment with CX-4945 strongly inhibited activation of AKT phosphorylation and glycometabolism. After treatment with CX-4945, it were observed that suppressed cell proliferation and cell migration and induced cell apoptosis. Thus, CK2 inhibitors, such as CX-4945, may provide a potential therapeutic target for patients with bladder cancer. Moreover, in vivo xenograft study showed that knockdown of CK2α decreased the tumorigenicity of bladder cancer cells.

## MATERIALS AND METHODS

### Clinical specimens and cell culture

Bladder cancer and adjacent non-tumor tissues were obtained from patients undergoing radical cystectomy at the First Affiliated Hospital of Nanjing Medical University. All patients provided signed informed consent, and the Research Ethics Committee of our institution approved this study. The specimens were immediately frozen and stored in liquid nitrogen. Pathological examination was performed to confirm cancer diagnosis.

Bladder cancer cell lines T24 and EJ were obtained from the Type Culture Collection of the Chinese Academy of Sciences (Shanghai, China). Cells were cultured in RPMI 1640 medium supplemented with 10% fetal bovine serum (FBS; Gibco, Australia) and 1% penicillin–streptomycin in an incubator with humidified 5% CO_2_ at 37°C.

All the bladder cancer specimens we used in our work are urothelial carcinomas. The cell type of T24, J82, EJ, 5637, 253J and TCC is Transitional Cell Carcinoma, while the cell type of RT4 is Transitional Cell Papilloma.

### Tissue microarray (TMA) and Immunohistochemistry (IHC)

TMA (16×10) was constructed from 160 cases of bladder tumor tissues. TMAs were kept at 4°C until they were ready for analysis.

Sections were arranged in duplicate cores per case. TMAs were treated with xylene and 100% ethanol, followed by decreased concentrations of ethanol. After antigen retrieval, TMAs were blocked and stained with antibodies against CK2α, followed by secondary antibody incubation and standard avidin biotinylated peroxidase complex method. Hematoxylin was used for counterstaining, and images were obtained with an upright microscope system (Nikon, JAPAN).

The total CK2α immunostaining score was calculated as the sum of the score for the proportion of positively stained tumor cells and the score for staining intensity given by two pathologists blinded to the clinical parameters. The proportion of positively stained tumor cells was scored as follows: “0” (<5%, negative), “1” (5%–25%, sporadic), “2” (25%–50%, focal), and “3” (>50%, diffuse). The intensity of staining was graded according to the following criteria: “0” (no staining), “1” (weak staining = light yellow), “2” (moderate staining = yellow brown), and “3” (strong staining = brown). The total immunostaining score, which ranged from 0 to 9, was calculated as the value of the proportion of positive cell score × staining intensity score. The expression level of CK2α was defined as follows: “−” (negative, score 0), “+” (weakly positive, score 1–3), “++” (positive, score 4–6), or “+++” (strong positive, score 7–9). Thus, CK2α protein expression in BCa specimens was divided into two groups: low CK2α expression group (CK2α“–” or CK2α“+”) and high CK2α expression group (CK2α“++” or CK2α“+++”) (Figure 2A).

### CK2α inhibitor

CK2α siRNA and control siRNA were purchased from Shanghai GenePharma Co., Ltd. Cells were seeded in a six-well plate at 10^^5^ cells/well 1 day before transfection, with a target of 30%–50% confluence at the time of transfection. Cells were transfected with 50 nmol/L siRNA using Lipofectamine RNAiMAX (Invitrogen) according to the manufacturer's protocol. Adequate inhibition of siRNA-mediated knockdown was confirmed by Western blot. The pcDNA3.1- CK2α or control pcDNA3.1-LacZ plasmid vectors were then transfected into T24 and EJ cells (0.5l g/mL in a 24-well plate) using Lipofectamine 2000 transfection reagent (Invitrogen), according to the manufacturer's protocol. Cells were harvested for qRT-PCR and Western blot or used in other assays at 48 h post-transfection.

Lentivirus constructs were generated to knockdown CK2α. The bladder cancer cell EJ was stably transfected with LV3pGLV-h1-GFP-puro negative control vectors (termed as shNC) and CK2α knockdown lentivirus (termed as shCK2α), following the manufacturer's instructions (Abm, Nanjing, China). Lentiviral constructs of CK2α knockdown were generated as previously described. Cells were plated in 6 wells dishes at 40% confluence and infected with the retroviruses. Meanwhile, polybrene (3 μg/ml) was added with the retroviruses to enhance infection efficiency. Stable pooled populations of bladder cancer cells were generated by selection using puromycin (3μg/ml) for 3 weeks. For CK2α knockdown cells, which achieved≥75% knockdown efficiency of mRNA was used for further studies.

CX-4945(small-molecule CK2 inhibitor) was purchased from Selleckchem.

### RNA extraction and quantitative RT-PCR

Total RNA from 44 frozen bladder tissues (22 for tumor tissues and 22 for paired adjacent normal tissues) and bladder cancer cells was extracted by Trizol according to the manufacturer's instructions. cDNA was prepared from 1 μg of total RNA in a 30 μL volume using PrimeScript^TM^ RT Master Mix (Takara Bio, Dalian, China). The reaction mixture was incubated at 37°C for 15 min, 85°C for 5 s, and 4°C for 5 min. The synthesized cDNA was used for PCR amplification or stored at −20°C for further analysis.

The PCR reaction was performed in a 25 μL volume containing 0.25 mM of each dNTP, 1 U HotstarTaq (Takara), and 0.5 mM of each primer (Takara): 5′-CCGAGTTGCTTCCCGATAC-3′ (forward) and 5′-GGGCTGACAAGGTGCTGAT-3′ (reverse) for CK2α; 5′-AGCGAGCATCCCCCAAAGTT-3′ (forward) and 5′-GGGCACGAAGGCTCATCATT′ (reverse) for β-actin.

### Western blot analysis

Cells were lysed, and the protein was extracted using RIPA buffer (Beyotime, China) and quantified by a BCA Protein Assay Kit (Beyotime, China). Equivalent quantities of protein were separated on 10% SDS–PAGE gels and transferred to polyvinylidene fluoride membranes. After blocking with 5% nonfat milk at room temperature for 1 h, the membranes were immunostained with primary antibodies at 4°C overnight, washed three times in TBST, and incubated with secondary antibody at room temperature for 1 h. Band signals were detected using a chemiluminescence system (Bio-Rad, USA) and analyzed using Image Lab Software. The following primary antibodies were used: primary antibodies against GLUT1, LDHA, LDHB, G6PD, 6PGD, and PKM2 were obtained from Epitomics, whereas those against HK1, HK2, AKT, and phospho-AKT(Ser473) were obtained from Cell Signaling Technology. CK2α was obtained from Millipore. The protein levels were normalized to β-actin (1:1000, Cell Signaling Technology).

### Measurement of glucose uptake

For glucose uptake experiments, cells were plated in six-well plates at a density of 3×10^5^ cells/well at 24 h before the experiment was performed. Cells were then washed with PBS, and uptake was initiated by incubating cells in 2 mL of Krebs–Ringer–HEPES (KRH) buffer (25 mMHepes, pH 7.4, 120 mMNaCl, 5 mMKCl, 1.2 mM MgSO_4_, 1.3 mM CaCl_2_, and 1.3 mM KH_2_PO_4_) containing 1 μCi of [3H]-2-deoxyglucose (PerkinElmer Life Sciences) for 20 min. Uptake was stopped by washing the cells with ice-cold KRH buffer. Cells were dissolved in 300 μL of lysis buffer (10 mMTris–HCl, pH 8.0, 0.2% SDS). Liquid scintillation spectrometry was performed to determine the radioactivity level of each aliquot. Disintegrations per minute value was used to evaluate the intracellular level of [3H]-2-deoxyglucose, and each assay was performed in triplicate.

### Measurement of extracellular lactate

Approximately 3×10^5^ cells/well were seeded into six-well plates that were incubated in 2 mL of RPMI 1640 with 10% FBS. After incubation for 24 h, the supernatant was collected. The lactate concentration in the supernatant was quantified by performing a fluorescence-based assay according to the manufacturer's instructions (L-Lactate Assay Kit, Cayman). The lactate concentration in the supernatant without seeded cells was also calculated and used to eliminate the effect of lactate on RPMI 1640 medium. Each assay was performed in triplicate.

### Cell viability assay

For cell viability assay, the transfected cells were seeded into 96-well plates at a density of 3000 cells/well. The CCK8 method was used to determine cell viability at 24, 48, 72, and 96 h after the cells were seeded. The absorbance was measured at 450 nm using a Tecan Infinite F200 microplate reader.

### Wound healing assay

Cells were seeded into six-well plates and cultured until they reached 95% confluence. A 200 μL pipette tip was used to generate a cross-shaped wound through the center of the well. The cultures were washed with PBS to remove cell debris, and the cells were incubated in RPMI 1640 medium without FBS. The wound was observed under a microscope (Olympus, Japan) at 40× magnification at two preselected time points (0 and 36 h), and the widths of wounds were counted.

### Apoptosis assay

After treatment with CX-4945 for 12 h, cells were trypsinized, stained with Annexin V and propidium iodide, and examined by flow cytometry.

### *In vivo* tumor xenograft studies

BALB/C female nude mice (4-6-weeks old, 18–22 g) were randomly divided into two groups (each containing 6 mice). The transfected EJ cells and controls were implanted with 1 × 10^7^ cells in 0.1 ml PBS per site on the axilla in nude mice (n=6). Tumor growth was monitored by measuring the width (W) and length (L) with calipers every 3 days, and the volume (V) of the tumor was calculated using the formula V=(W^2^×L)/2. After 21 days, mice were sacrificed and checked for final tumor size. At the end of the experiment, the tumors were removed and fixed in 4 % formalin for immunohistochemical analysis. The animal studies were performed in accordance with the institutional ethics guidelines for animal experiments.

### Statistical analysis

Statistical analysis was performed using SPSS 20.0 and GraphPad Prism 5. All data are presented as the mean ± standard deviation. Differences between two groups were analyzed using Student's *t*-test. Cell viability assay and in vivo tumor xenograft results were assessed by repeated measures ANOVA. P < 0.05 was considered statistically significant. All experiments were repeated more than three times, and each experiment was performed in triplicate.

## CONCLUSIONS

Our study revealed the relationship between CK2α overexpression and unfavorable progression in bladder cancer patients. For the first time, we reported that targeting CK2 suppressed bladder cancer cell survival via downregulation of AKT-mTOR and the glucose metabolic pathway in urinary bladder cancer. However, the precise mechanism needs to be elucidated. Our results could be regarded as the basis for a candidate prognostic biomarker and new potential therapeutic strategies for bladder cancer. Our study also provided new insights into the regulation of glycolysis by protein kinase CK2.
